# Personal experience with abortion: a determining factor in Physician's attitudes towards abortion

**DOI:** 10.1016/j.xagr.2025.100478

**Published:** 2025-03-20

**Authors:** Reagan M. Ingoma, So Yoon Kim, Erick N. Kamangu

**Affiliations:** 1 College of Medicine (Ingoma), Yonsei University, Medical Ethics and Law, researcher, Seoul/Korea; 2 Centre Hospitalier Universitaire Renaissance (Mokeke), Kinshasa, DR Congo; 3 College of Medicine, Medical Law and Ethics (Kim), Professor at Yonsei University, Seoul/Korea; 4 Molecular Biology Service, Department of Basic Sciences (Kamangu), Faculty of Medicine, University of Kinshasa, Kinshasa, Democratic Republic of Congo

**Keywords:** abortion, attitude, physicians, Kinshasa

## Abstract

**Background:**

The increase in illegal abortions raises concerns about the role of physicians in providing safe abortion care. Understanding physicians' views in multicultural countries like the DRC is essential, even though the Maputo Protocol permits safe abortion care. The research aims to determine the impact of their experience with abortion on their attitudes towards abortion.

**Method:**

This is cross-sectional study involving 265 physicians from The Democratic Republic of Congo, conducted between April 1st and June 30th, 2024, using a convenience sampling technique that determines doctors' attitudes toward abortion to lay the groundwork and pave the way for future research on this issue. The study used a chi-square test to analyze categorical variables, Spearman correlation with 2 hypotheses, and multinomial logistic regression to predict the connection between the explanatory, the confounding variables, and the physician attitudes towards abortion.

**Results:**

On a scale of 1−5, the median score was 3, the mean was 2.95, and the standard deviation was 0.661. We found a significant correlation between physicians' personal experiences with abortion and their attitudes toward the practice (r_s_ 0.211, *P*.001). Doctors who have personally experienced abortion are more likely to support abortion rights than pro-life doctors (OR: 6.52, *P*.005).

**Conclusion:**

Targeted training programs for healthcare providers on the medical, legal, and ethical aspects of abortion care are crucial for equipping them with essential knowledge and skills. Public health initiatives should create and share standardized protocols for abortion care to ensure consistent quality across all healthcare facilities, especially in rural and underserved areas, promoting equitable access to such services.

**AJOG Global Reports at a Glance**

**Why was the study conducted?**

It was crucial to understand how those responsible for providing care perceive abortion and what determinants shape their perceptions.

**Key findings**

The study found a significant correlation between physicians' personal experiences with abortion and their attitudes toward the practice (rs 0.211, *P.*001). Doctors who have personally experienced abortion are more likely to support abortion rights than pro-life doctors (OR: 6.52, *P*.005).

**What does this study add to what is already known?**

Studies on abortion in the Democratic Republic of Congo (DRC) mainly analyze data related to the general population, including clinical, para-clinical, and sociodemographic information. The present study demonstrates the correlation between a physician's previous experience with abortion and their current stance on the issue in the DRC.

## Introduction

The 6.2 million abortions in sub-Saharan Africa show that the problem remains alarming.^1^ The abortion rate in Kinshasa, the bustling capital city of the Democratic Republic of Congo (DRC), witnessed a significant surge. Between 2016 and 2021, the rate ascended from 56 abortions per 1000 women^2^ of childbearing age to a striking 105.3 per 1000,^3^ marking a noteworthy 94% increase over 5 years. Abortion is a contributing factor in approximately 7.9% of maternal deaths in sub-Saharan Africa.^4^ This surge in abortion rates sheds light on the pressing need to address the intricate public health implications associated with abortion practices within the DRC.^5^ The rise in abortions is due to factors like societal dynamics, economic conditions, cultural influences, inadequate access to sexual and reproductive health services, lack of contraception knowledge, and abortion stigma.^6^

Physicians perform abortions, providing preabortion counseling, prioritizing patient comfort, and ensuring smooth recovery post-abortion. They prioritize patient well-being, empathy, and expertise in the procedure.^7^ Accurate information from physicians empowers women and girls to make informed reproductive health choices, highlighting education's role in decision-making.^8^ Upholding the principles of patient consent and confidentiality, physicians create a safe and welcoming healthcare environment where trust and support thrive.

Various factors, including personal and religious beliefs, influence physicians' attitudes toward abortion. These beliefs can lead some healthcare providers to refuse abortion services, creating disparities in access to reproductive healthcare, particularly in regions with few professionals or solid religious influences.^9^ More positive opinions toward abortion are typically the consequence of more training and clinical experience in abortion treatment. A thorough education and practical training in abortion care increase the likelihood that medical students and healthcare professionals would support the provision of these services.^10^

Physicians' personal experiences with abortion can shape their perspectives, leading to greater understanding and support for patients. Individuals who have experienced or know someone who has undergone an abortion tend to show greater compassion and empathy, improving patient care in this sensitive area.^11^ The work environment significantly influences health professionals' abortion attitudes, with supportive environments promoting respect for reproductive rights, leading to more compassionate and nonjudgmental care, ultimately improving access to safe and respectful abortion services.^12^

Being subjected to prejudice and stigma around abortion can hurt attitudes.^13^ Healthcare professionals who operate in settings where abortion is stigmatized may get jaded, which may have an impact on the standard of care they offer.

The World Health Organization (WHO) has recommended that member states eliminate the legal obligation on healthcare providers, including doctors, to report women who have undergone abortions.^14^

Most countries with legalized abortion have distinct regulations that set it apart from other healthcare systems.^15^ They still consider abortion a crime, punishing those who have abortions and those who provide abortion services or help with getting an abortion. Sharing information about abortion can also lead to penalties.

The DRC signed the Maputo protocol, which allows physicians to perform medical abortions in cases of sexual assault, rape, incest, and when the pregnancy poses a risk to the mental or physical health of the mother or the life of the mother or fetus.^16^ The DRC's authorities are implementing Article 14, Paragraph 2c of the Maputo Protocol, allowing safe abortions despite no amendments to the penal code. However, abortions performed by untrained personnel or in uncertified facilities are deemed clandestine and face legal penalties.^17^

Medical practitioners cannot offer abortion services except under specific conditions. They may, therefore, face legal consequences. This situation illustrates concerns about the persistent prevalence of clandestine abortions despite the adoption of the Maputo Protocol. In the DRC, abortion in 2021 mainly happened because women had short gaps between pregnancies (23.8%) or because they did not have enough money (21.0%).^18^

Studies on abortion in the DRC mainly analyze data related to the general population, including clinical, para-clinical, and sociodemographic information. However, it is crucial to understand how those responsible for providing care perceive abortion and what determinants shape their perceptions. It is essential to study the connection between a person's past abortion and their views on abortion among doctors in the DRC to answer the following question: Does a physician's attitude toward abortion depend on whether he or she has had an abortion or knows someone close to him or her who has had an abortion? This approach will help test these hypotheses: (1) a personal experience with abortion is significantly linked to a positive attitude towards abortion among doctors, (2) a personal experience with abortion is significantly linked to a negative attitude toward abortion among doctors, (3) There is no significant association between physician attitudes toward abortion and their personal experience with abortion.

## Method

### Data source

The study used a cross-sectional descriptive design. 265 physicians, regardless of their specialties from Kinshasa, the capital of the DRC, recruited through a convenience sampling technique, freely answered an online self-administered questionnaire about their attitudes toward abortion between April 1st and June 30th, 2024. Convenience samples are frequently used in behavioral researches.^19^^,^^20^ While the number of respondents may not be representative of the total number of doctors in the study areas, we were less concerned with representativeness than with assessing the elements that determine doctors' attitudes toward abortion to lay the groundwork and pave the way for the future research on this issue. The respondents completed 2 parts of the questionnaire. The first part concerned the social demographic data of the participants, and the second part explored the legal considerations of the availability of abortion services and care based on the current legal framework in the DRC as enacted from the Maputo Protocol on safe abortion. The questionnaire was initially administered in French and subsequently translated into English for interpretation by a qualified translator.

**Fig. 1:**
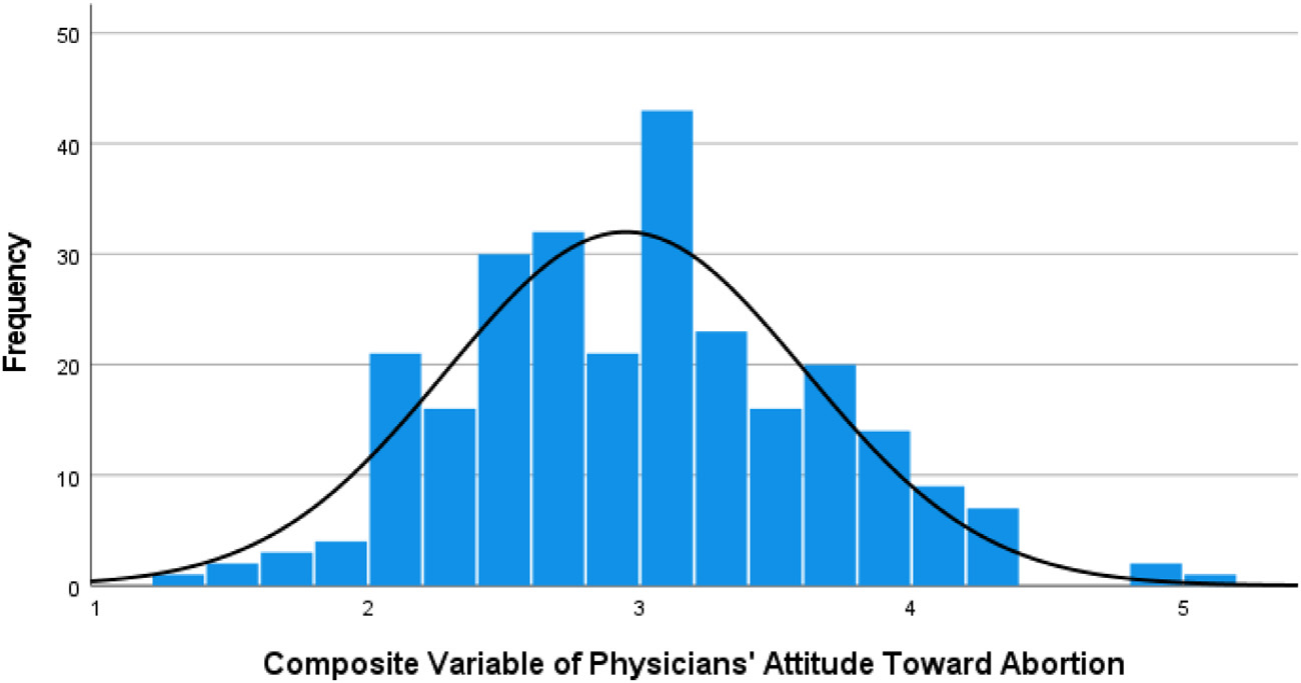
Mean, Median, and standard deviation of the composite variable of physicians' attitude on abortion

Attitude scores were measured from 1 to 5, with 1 indicating strong disapproval and 5, strong approval of the statement. We tested the validity and reliability of the questionnaire proceeding with the survey. Additionally, a pilot study was conducted with a small sample group (30 participants) prior to the main study to identify any ambiguities or issues with the Likert scale items and ensure that the questions were clear and understandable. We selected 10 out of 16 original items, achieving an acceptable Cronbach's Alpha score of .787.^21^ (Fig. 1).

Ingoma. Personal experience with abortion. Am J Obstet Gynecol 2025.

### Dependent variable

In this study, we gauged Abortion Attitudes using a 10-item Likert scale. We computed a composite variable by calculating a median score of 3, establishing that scores above 3 are pro-choice, below 3 are pro-life, and precisely 3 are undecided. This approach clarifies respondents' attitudes, mirroring methods from previous research on abortion attitudes.^22^

### Explanatory variable

The independent variable is personal experience with abortion, which refers to a physician's personal experience with abortion. Personal experience with abortion is related to whether the respondents have had an abortion themselves or know someone close to them (spouse or relatives) who has had an abortion. The variable was dichotomized respectively into Yes or No.

### Confounding factors

We considered various confounding factors that might affect the results. These factors identified in previous studies include age and religion,^23^ gender, marital status, education,^24^ the level of institution, and work experience. The following table highlights the operational definition of each confounding variable (Table 1).

**Table 1:** Operational definition of the independent variables

Variable	Definition
1. Current age (in years)	During the survey, we categorized the respondents' ages into 5 classes based on the minimum age for graduating as a doctor in the DRC.
	− 25−34
	− 35−44
	− 45−54
	− 55−64
	− 65 and over
2. Gender	Respondents had to specify whether they were males or females
3. Level of Education	Depending on his specialty, the physician classified himself as a generalist or a specialist based on his level of education.
4. Level of institution	Refers to the level of healthcare facility in which the respondent works. According to the DRC regulation, there are 3 levels of health facilities classified as fellow;
	− Primary
	− Secondary
	− Tertiary
5. Marital status	Marital status was a dichotomous variable with:
	− Married for current married respondents, and
	− Single for single, divorced, widow respondent, or any other status other than married
6. Religion	Refers to the fact that the respondent currently practices religion. They were categorized into:
	− Catholic:
	− Protestant
	− Revival Church
	− Islam
	− Others (Kimbanguiste, traditional, no religion, animist…)
7. Work experience	Respondents were asked how long they had been working as physicians in the DRC. The responses were categorized into 3 groups.
	− Low: 1−5 years
	− Middle : 6−10 years
	− High : 11 years and over

### Statistical analysis

Descriptive statistics were analyzed using IBM SPSS Statistics 28.0.0.0, generating frequency distributions and measures such as mean and standard deviation. A chi-square test with *P*<.05 assessed categorical independent variables' effects on physicians' attitudes toward abortion. Due to the non-normal distribution (Kolmogorov-Smirnov test *P*<.001), the Spearman correlation analyzed personal experience influences. Multinomial logistic regression models predicted connections between variables and abortion attitudes, maintaining *P*<.05 significance.

## Results

On a scale of 1−5 regarding the composite variable of abortion attitude, the median score was 3, the mean was 2.95, and the standard deviation was 0.661. Respondents generally showed a pro-life or undecided attitude in most cases, indicating a relatively tight clustering of responses around the average (Table 2).

**Table 2:** Multi-item statements to measure physicians' attitude toward abortion (n = 265)

	Attitude							
	Strongly disagree	disagree	undecided	agree	Strongly agree			
Items	n (%)	n (%)	n (%)	n (%)	n (%)	Mean	Median	SD
Abortion should be legal in DRC	109(41.1)	65(24.5)	24(9.1)	42(15.8)	25(9.4)	2.28	2	1.38
Abortion should be legal in cases of incest	66(24.9)	53(20)	24(9.1)	73(27.5)	49(18.5)	2.95	3	1.49
Abortion should be legal in cases of rape	65 (24.5)	47(17.7)	30(11.3)	76(28.7)	47(17.7)	2.97	3	1.47
Abortion should be equally available regardless of income	76(28.7)	55(20.8)	36(13.6)	68(25.7)	30(11.3)	2.7	3	1.41
Abortion should be legal if birth control fails	87(32.8)	80(30.2)	34(12.8)	53(20)	11(4.2)	2.32	2	1.38
Abortion should be legal when the mother's health is in danger	7(2.6)	2(0.8)	3(1.1)	122(46)	131(49.4)	4.39	4	0.79
Abortion should be available in primary clinics	68(25.7)	56(21.1)	18(6.8)	86(32.5)	37(14)	2.88	3	1.45
A fetus should have legal rights	15(5.7)	17(6.4)	24(9.1)	99(37.4)	110(41.5)	4.03	4	1.13
The law has no right to tell a woman what to do with her body	88(33.2)	80(30.2)	26(9.8)	45(17)	26(9.8)	2.4	2	1.36
Abortion should be entirely the women's decision	100(37.7)	82(30.9)	25(9.4)	37(14)	21(7.9)	2.23	2	1.3

Note: 1. Strongly disagree, 2. Disagree, 3. Undecided, 4. Agree, 5. Strongly agree

Personal experience with abortion showed a statistically significant distribution among respondents. 61.3% of doctors who had never had an abortion or knew someone close to them who had an abortion were in favor of pro-life. On the other hand, those who had experience with abortion were more uncertain, with 51.8% being undecided.

The average age of respondents was 38.7 years. Among those aged 25−34, 50% were pro-life, 42% were undecided, and only 6% were prochoice. Among physicians aged 35−44, 47.7% were prolife, 8.3% were prochoice, and others were undecided. Most categories showed a trend toward being pro-life or undecided, but it was not statistically significant.

48.2% of female doctors were pro-life versus 49.4% of men, while 3.5% of female respondents and 8.9% of men were pro-choice.

In 8.6% of cases, general physicians were pro-choice, while only 2.9% of specialists showed a pro-choice attitude. However, in both categories, 49.2% of general physicians and 48.5% of specialists were pro-life.

Single and married physicians show similar views on abortion, with 49.4% pro-life and 8.9% pro-choice among single physicians and 48.9% pro-life and 6.5% pro-choice among married physicians. Most physicians are either pro-life or undecided, with only 4.8% to 9.3% identifying as pro-choice. Notably, 46.4% of secondary and 41.6% of tertiary hospitals remain undecided, highlighting the uncertainty among healthcare workers.

Some 80.7% of respondents identified as Catholic, Protestant, or revivalist, with only 7.9% holding pro-choice views, while most held pro-life views. Muslim physicians and others of different faiths showed the same pattern (Table 3)

**Table 3:** Bivariate analysis of general characteristics of the respondents

	Abortion attitude					
	Pro-life		Undecided		Pro-choice	
General characteristics of the respondents	n	%	N	%	n	%
**Current age (in years)**						
25−34	38	50	32	42.1	6	7.9
35−44	62	47.7	59	44.7	11	8.3
45−54	21	48.8	20	46.5	2	4.7
55−64	7	58.3	5	41.7	NA	NA
65 and over	2	100	NA	NA	NA	NA
**Gender**						
Female	41	48.2	41	48.2	3	3.5
Male	89	49.4	75	41.7	16	8.9
**Personal experience with abortion**^a^						
No	76	61.3	43	34.7	5	4
Yes	54	38.3	73	51.8	14	9.9
**Level of education**						
Generalist	97	49.2	83	42.1	17	8.6
specialist	33	48.5	33	48.5	2	2.9
**Marital status**						
Single	39	49.4	33	41.8	7	8.9
Married	91	48.9	83	44.6	12	6.5
**Work experience**						
Low	38	45.2	42	50	4	4.8
Middle	52	54.7	36	37.9	7	7.4
High	40	46.5	38	44.2	8	9.3
**Level of institution**						
Primary	15	55.6	11	40.7	1	3.7
Secondary	60	48	58	46.4	7	5.6
Tertiary	55	48.7	47	41.6	11	9.7
**Religion**						
Catholic	32	51.6	24	38.7	6	9.7
Protestant	38	55.1	27	39.1	4	5.8
Revival church	36	43.4	40	48.2	7	8.4
Islam	3	60	2	40	NA	NA
Others	21	45.7	23	50	2	4.3
Total (n=265)	130	100	116	100	19	100

Chi-square test is significant at: ^a^P-value <.05; ^b^P-value <.01; ^c^P-value <.001.NA, Not applicable.

The study revealed a modest yet notable association between doctors' personal experiences of having had an abortion themselves or knowing someone close to them (spouse or relatives) who has had an abortion and their attitude on the subject of abortion (r_s_: .0211, *P.*001). Doctors who have had personal experiences with abortions may have their perspectives shaped by those encounters. This finding shows that individual medical experiences can broadly influence their professional viewpoints. This study opens new avenues for understanding the intricate interplay between personal experience and professional perspective in the medical field (Table 4).

**Table 4:** Correlation between physicians' personal experience and their attitude toward abortion

			Abortion attitude
Spearman's rho	Personal experience with abortion	Correlation Coefficient	.211^a^
		Sig. (2-tailed)	0.001
		N	265

a Correlation is significant at the 0.001 level (2-tailed).

Doctors who have undergone personal experiences with abortion are significantly more likely to advocate for abortion rights, with a 6.52 times higher likelihood (*P*.005) compared to those who have not had such experiences. The fact confirms the same trend, with there being 2.6 times more undecided individuals than pro-life in comparison to those who have never undergone abortion (*P*<.001).

Older respondents are more pro-life (OR 0.282, *P*.017), while younger doctors lean towards being pro-choice or undecided compared to older counterparts (OR 0.875, *P*.573Women show less tendency than men to support a pro-life stance (OR 0.299, *P*.088) and may be undecided rather than pro-life (OR 1.152, *P*.623).

Findings indicate that medical practitioners with low or intermediate experience (OR 0.149, *P*.067, and OR 0.241, *P*.047, respectively) are more likely to support a woman's right to choose. Increased experience correlates with this tendency, but many remain undecided on abortion. Single physicians are nearly twice as likely to exhibit a pro-choice attitude as married physicians (OR 1.928, *P*.37) (Table 5).

**Table 5:** Association of predictors with attitude toward abortion attitude among physicians

	Pro-choice (vs Pro-life)	Undecided (vs Pro-life)	Undecided (vs Pro-choice)
	Model 1	Model 2	Model 3
Predictors	OR	OR	OR
**Current age (in years)**			
Ref. 65 and over	0.282^a^	0.875	3.101^a^
**Age group**			
25−34	1238226720	132946023.157^c^	5.97.10^−9^
35−44	644343382.6	178617320.833^c^	1.15.10^−8^
45−54	144900803.6	146279181.541^c^	5.10.10^−8^
55−64	9.73	125391762.4	0.103
65 and over	1	1	
**Gender**			
Female	0.299	1.152	3.85
Male	1	1	
**Personal experience with abortion**			
Yes	6.52^b^	2.60^c^	0.399
No	1	1	
**Level of education**			
Generalist	3.316	0.814	0.302
specialist	1	1	
**Marital status**			
Single	1.928	0.727	0.377
Married	1	1	
**Work experience**			
Low	0.149	1.637	12.875^a^
Middle	0.241^a^	0.83	3.176
High	1	1	
**Level of institution**			
Primary	0.094	0.622	6.582
Secondary	0.371	0.934	2.519
Tertiary	1	1	
**Religion**			
Catholic	1.954	0.651	0.315
Protestant	1.459	0.706	0.478
Revival church	2.923	0.995	0.343
Islam	9.02. 10^−8^	0.67	16837900
Others	1	1	

Multinominal logistic regression is significant at:

a P-value <.05

b P-value <.01

c P-value <.001. 95% Confidence Interval.

The views of religious physicians of different denominations on the issue of abortion may vary. Findings indicate that doctors from Catholic, Protestant, or revivalist churches may be more inclined to support abortion rights, while those from traditional churches may be more opposed to abortion. Each denomination's theological teachings and interpretations may influence these differences in perspective. Conversely, Muslim doctors were more likely to be pro-life than pro-choice than those in the reference group.

## Discussion

The overall finding showed a mostly pro-life and undecided attitude among physicians, indicating a relatively tight clustering of responses around the average, suggesting that while most respondents leaned towards a neutral or slightly pro-life stance, a notable segment of individuals held more definitive views. A closer examination of the data reveals that a significant majority of participants expressed either a pro-life position or indicated that they were undecided on the issue. These results may be indicative of broader societal influences, including cultural background, religious beliefs, and personal experiences, which often shape individuals' perspectives on abortion.^25^^,^^26^ Furthermore, the observed variance in responses indicates that while there is a substantial group adhering to these attitudes, there exists a minority that feels strongly in favor of pro-choice perspectives. Following previous studies, medical reasons for abortion were well-received by physicians in the USA.^27^ Physicians widely recognized the medical justifications for allowing abortions, understanding the potential risks to the health and well-being of the pregnant individual.^28^ Medical professionals recognize the need for safe, legal access to abortion when the health or life of a pregnant person is at risk. They support compassionate, evidence-based decisions and recognize the importance of informed discussion on this polarizing issue. Understanding diverse viewpoints promotes empathy and critical thinking. Physicians are encouraged to consider the complexities behind diverse opinions on reproductive rights.

A personal experience with abortion is associated with more favorable attitudes toward abortion among physicians compared to those without such a history. The relationship between personal experience and views on abortion rights is profound.^27^ Doctors who first witnessed the reality of this issue occupy a unique position. A striking fact emerges when examining their position on abortion about their personal history with the procedure. Doctors who have a personal experience with abortion are 6.52 times more likely to defend abortion rights than their opponents. This substantial statistically significant increase underlines the significant impact of direct personal experience on a person's point of view. The phenomenon is not limited to those firmly taking a stance. An equally compelling pattern emerges among those who find themselves undecided. Physicians who have personally had an abortion are significantly 2.6 times more likely to be undecided rather than identifying as pro-life compared to their counterparts without such an experience, which speaks volumes about the complex interplay of personal history and medical ethics. Physicians with a personal experience with abortion may have undergone the emotional, psychological, and physical aspects of the procedure. Individuals with personal experience may demonstrate greater empathy and understanding towards patients seeking abortion, which can foster a more supportive approach. Past research has suggested that stigma can shape the health professional attitude toward abortion.^29^ Doctors who have had an abortion may better understand the stigma and difficulties related to it. This awareness could encourage them to have a more positive attitude towards abortion, reduce the stigma, and support others in the same situation.

Doctors with personal abortion experience offer valuable insights into the medical and ethical aspects of the procedure and emphasize the role of healthcare professionals in increasing access to a safe abortion.^30^ In Jamaica, healthcare providers tend to become more empathetic and supportive towards abortion when they have personal experiences or know someone who has gone through it.^31^ These trends are consistent with our findings, suggesting a significant positive correlation between physicians' experience and their attitudes toward abortion. By drawing on their own experiences, these physicians can gain a deeper understanding of the importance of safe and accessible abortion services, which in turn can lead to a significantly more supportive attitude towards this vital healthcare option.

Healthcare providers viewed abortions differently in South Africa. They believed that a medical abortion was the woman's responsibility, so she, not the nurse or doctor, would answer to God for her choices.^32^ These findings contrast with our results, where religion plays a crucial role in physicians' attitudes toward abortions. This differing view may stem from the fact that most of the people interviewed for our study were Christians, who generally believe in the sanctity of life from the embryonic stage onwards, regard abortion as a sin, and believe that only God can decide on the future of a human being.

In The USA, Pro-choice were less likely to expect an increase in laws restructuring abortion.^33^ Medical practitioners are obligated to provide medical assistance in abortions due to the ethical obligation of prioritizing patient autonomy and well-being, which is the reason for being pro-choice. This duty to support patient choice is especially significant in the context of reproductive health, where the implications of denying care can lead to severe physical, emotional, and psychological consequences for individuals seeking abortions. Moreover, understanding reinforces the pro-choice stance that access to safe and legal abortion services is a critical component of comprehensive healthcare. Numerous studies have shown that when access to abortion is restricted, the rates of unsafe procedures increase,^34^, ^35^, ^36^ leading to higher morbidity and mortality rates among individuals who are unable to obtain the care they need. Thus, medical practitioners, often on the front lines of reproductive health, recognize their role in advocating for policies that protect and expand access to these essential services rather than withholding them based on personal beliefs or biases. In addition, the reproductive rights landscape is continually evolving, shaped by legal, social, and scientific advancements.^37^ Physicians must understand the implications of being pro-choice on individual rights, public health, social justice, and gender equality. Supporting individuals' right to make informed reproductive health decisions. Acknowledging medical professionals' responsibilities and ethical principles can promote a more compassionate dialogue on this critical issue. Moreover, Legal, cultural, and logistical barriers limit access to reproductive health services, including safe abortion care, in the DRC. Insufficient training for healthcare professionals and stigma hinders open patient dialogue, compromising care quality and leading to adverse health outcomes for women.

While the Maputo Protocol established the DRC government's commitment to enhance access to safe abortion as outlined by its terms, deeply rooted social norms and sanctions may hinder the actual impact.^38^ The persistence of social stigma and fear of rejection likely influenced participants' attitudes and shaped their pathways of information-gathering. The predominance of pro-life attitudes among those with personal abortion experiences raises complex questions. It may reflect the internalization of societal pressures and fear of judgment, which are deeply rooted in the local socio-cultural fabric. While our study provides initial insights, it underscores the urgent need to address societal stigma as part of comprehensive approach. This observation underscores the complex interplay between legal, social, and personal factors in shaping abortion attitudes, highlighting the importance of addressing societal stigma as part of comprehensive reproductive health policies.

A study from Colombia highlights a prevailing belief among physicians that equates abortion with murder although the constitutional court partially decriminalized abortion since 2006.^39^ This perception leads physicians to prioritize the fetus's rights over the woman's physical risks from continuing the pregnancy.^39^ The context of the DR Congo is particular. 78.9% of respondents believe fetuses should have legal rights, including the right to life. Nevertheless, an overwhelming 96% expressed compassion and support for abortion in cases where a woman's life is in danger. This dichotomy highlights the complex interplay between legal, moral, and cultural beliefs and the realities faced by women in crises, highlighting the importance of policies that balance the rights of the fetus with the health and autonomy of the woman.

In light of these challenges, it is crucial to promote training programs that cover clinical and psychosocial aspects of abortion care, helping healthcare providers support patients respectfully. Improving access to accurate information on family planning and reproductive rights is vital for informed decision-making.

## Strengths and Limitations

The study sample may not represent all physicians, but it provides valuable information about the observed characteristics and trends. This insight can lay the groundwork for future research, allowing for the exploration of a more comprehensive array of healthcare professionals, leading ultimately to a deeper understanding of the subject and its significance for healthcare practices.

Although a considerable portion of respondents had personal experiences with abortion, a mere 9.9% of those individuals adopted a pro-choice perspective, suggesting that legal, social, or personal factors may play a crucial role in shaping the beliefs of those who identify as pro-life or undecided. Investigating the factors that shape pro-life attitudes toward abortion among physicians with abortion experience is essential for understanding the dynamics within this specific subgroup of respondents. However, the current study does not offer this analysis, exposing the research to a potential bias. Future research should investigate the factors underlying this trend to understand these attitudes and their possible implications.

We acknowledge that using a convenience sampling technique in this study limits the generalizability of the findings to all physicians in Kinshasa. The sample may exhibit a bias towards individuals who are more accessible or willing to participate, thereby potentially omitting specific subgroups within the medical profession. To assess if our sample reflects the broader demographic and professional distribution of physicians in Kinshasa, a comparison with known demographic data from professional medical associations or census data could provide insight. Unfortunately, such comparative data was unavailable for our study, meaning that the extent of any bias introduced by our sampling method remains uncertain. Future studies should employ random sampling techniques to ensure a more representative sample of the physician population in Kinshasa.

## Conclusion

Personal experiences with abortion can affect physicians’ attitudes, highlighting insufficient access and quality of abortion care in the DRC health system. Targeted training programs for healthcare providers on the medical, legal, and ethical aspects of abortion care are crucial for equipping them with essential knowledge and skills. Public health initiatives should create and share standardized protocols for abortion care to ensure consistent quality across all healthcare facilities, including rural and underserved areas, promoting equitable access to such services. Additionally, fostering a supportive environment where healthcare providers can discuss and address their concerns and attitudes toward abortion without fear of judgment or retribution is crucial.

## CRediT authorship contribution statement

**Reagan M. Ingoma:** Writing – original draft, Investigation, Formal analysis, Data curation, Conceptualization. **So Yoon Kim:** Validation, Supervision, Project administration, Methodology. **Erick N. Kamangu:** Writing – review & editing, Validation, Supervision, Methodology, Data curation.
